# A first case of ductal adenocarcinoma of the prostate having characteristics of neuroendocrine phenotype with *PTEN*, *RB1* and *TP53* alterations

**DOI:** 10.1186/s12920-021-01093-9

**Published:** 2021-10-09

**Authors:** Hiroaki Kobayashi, Takeo Kosaka, Kohei Nakamura, Kazunori Shojo, Hiroshi Hongo, Shuji Mikami, Hiroshi Nishihara, Mototsugu Oya

**Affiliations:** 1 Department of Urology, Keio University School of Medicine, 35 Shinanomachi, Shinjuku-ku, Tokyo, 160-8582 Japan; 2 Genomics Unit, Keio Cancer Center, Keio University School of Medicine, Tokyo, Japan; 3 Division of Diagnostic Pathology, Keio University School of Medicine, Tokyo, Japan

**Keywords:** Ductal adenocarcinoma, Neuroendocrine prostate cancer, Next-generation sequencing, Case report

## Abstract

**Background:**

Ductal adenocarcinoma and neuroendocrine cancer are rare subtypes of prostate cancer with poor prognosis and limited therapeutic options. We present the first case of ductal adenocarcinoma having a neuroendocrine phenotype.

**Case presentation:**

A 63-year-old man presented with gross hematuria and urinary retention, and his serum prostate-specific antigen level was 4.58 ng/mL. We performed transurethral resection of the prostate, and the diagnosis was ductal adenocarcinoma with a Gleason score of 5 + 4 for acinar adenocarcinoma. Magnetic resonance imaging showed local invasion of left lobe of the prostate and bone metastasis of the left trochanteric section of the femur. Multidisciplinary treatments such as androgen deprivation therapy, chemoradiation therapy, and surgery for metastatic lesions have led to long-term survival. Since next-generation sequencing revealed *PTEN* and *RB1* co-loss and *TP53* mutations, we re-evaluated the immunohistochemistry and he was found to be positive for synaptophysin.

**Conclusions:**

This is the first Japanese case of ductal adenocarcinoma with a neuroendocrine phenotype. Genetic analysis may help not only guide the therapeutic strategies, but also sometimes with the diagnosis.

## Background

Ductal adenocarcinoma (DCa) of the prostate is a rare morphologic subtype, occurring in less than 1% and up to 5% in cases of acinar adenocarcinoma (ACa), defined histologically by elongated cells and papillary or cribriform architecture [1–3]. Since DCa often presents at an advanced clinical stage with local or distant metastases and no effective treatment has been established, the prostate-specific mortality is significantly worse than that of ACa [1]. In contrast, neuroendocrine prostate cancer (NEPC) is an extremely rare entity with poor prognosis and limited therapeutic options [4]. The pathological characteristics of NEPC include staining with immunohistochemical markers (CD56, synaptophysin, chromogranin A, and NSE), high proliferative rate (Ki67 > 50%), and the presence of TMPRSS2-ERG rearrangement. Synaptophysin is the most sensitive marker, NSE is also sensitive but not specific, and chromogranin A is the most specific marker [5, 6]. Little is known regarding the genetic and specific histopathological characteristics of DCa and NEPC, and to date, few studies have investigated the genetic profile of these tumors. Therefore, the accumulation of case reports and genetic or histopathological analyses is expected to add to our understanding to this disease entity. Herein, we report a case of a DCa mixed with ACa with a neuroendocrine phenotype.

## Case presentation

**Fig. 1:**
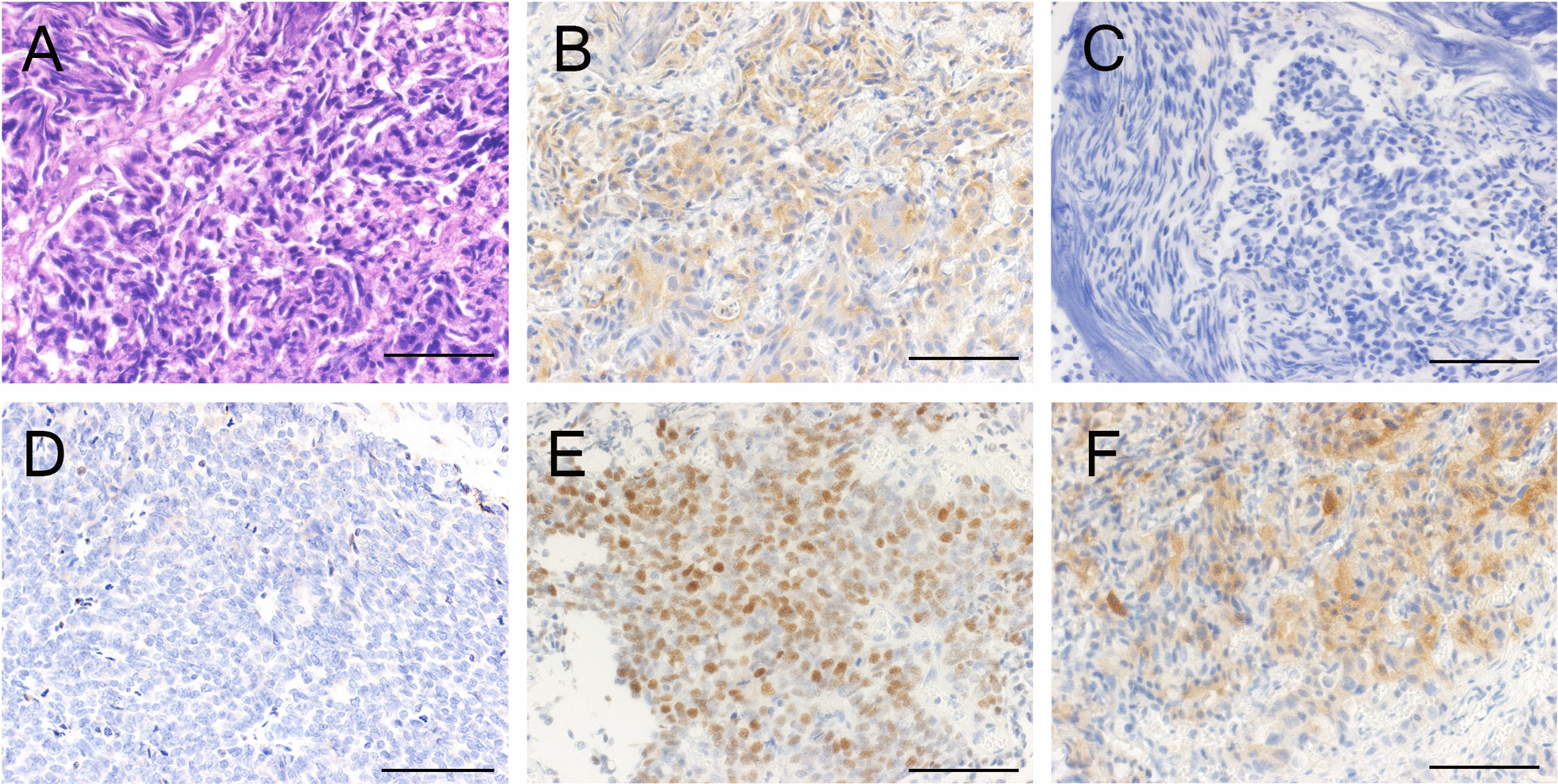
Representative images of **A** hematoxylin and eosin staining and **B** PSA, **C** loss of PTEN, **D** loss of RB1, **E** TP53 and **F** synaptophysin immunohistochemical staining of transurethral resection of prostate specimens. Bar = 0.1 mm

**Fig. 2:**
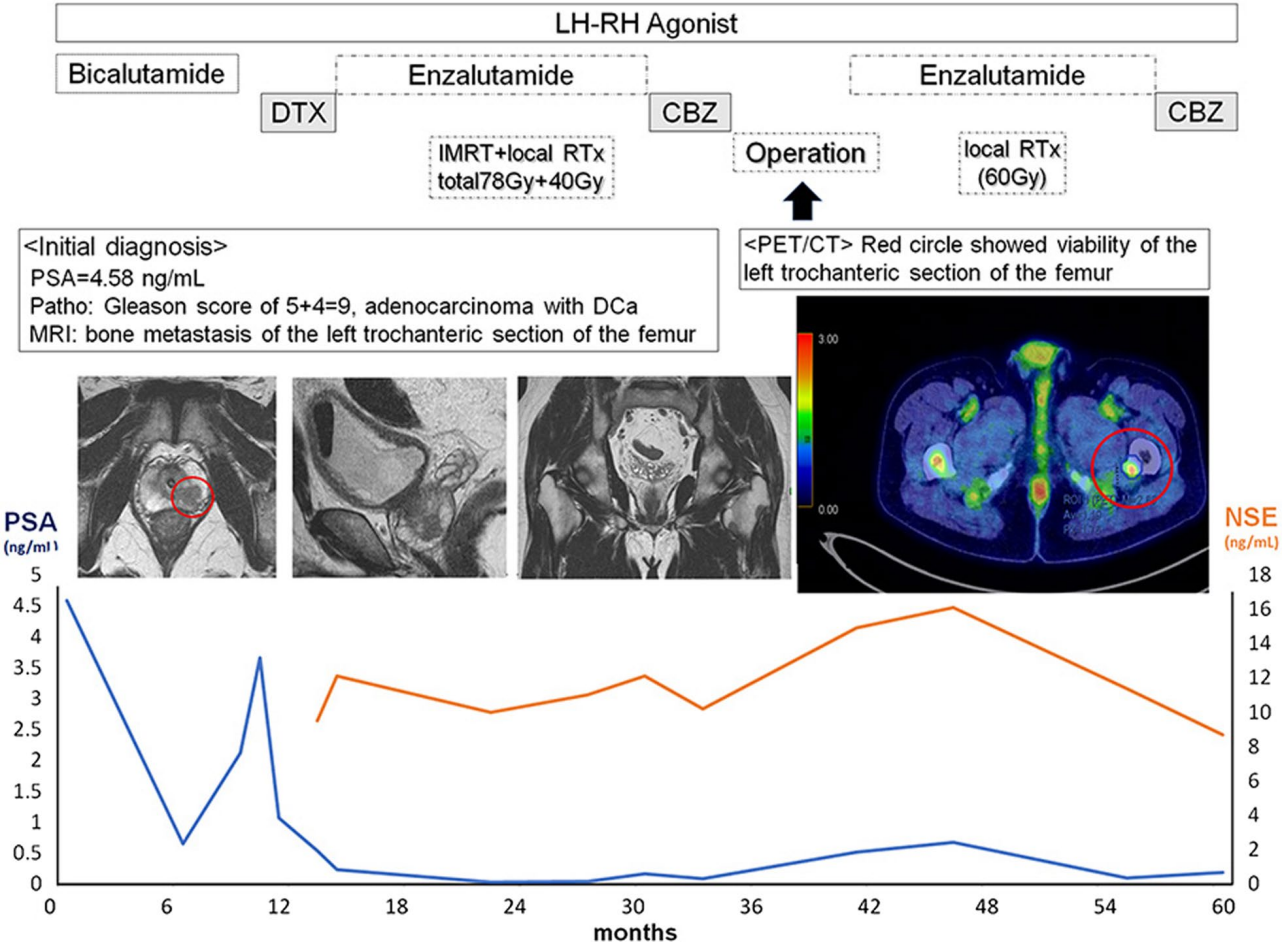
Serum PSA and NSE levels and time course for multidisciplinary treatments

A 63-year-old man presented with gross hematuria and urinary retention. He had no remarkable medical or family history. His serum prostate-specific antigen (PSA) was 4.58 ng/mL, and urine cytology was class 3 (atypical urothelial cells). Since cystoscopy revealed a papillary tumor in the prostatic urethra, we performed transurethral resection of the prostate. The pathological diagnosis was poorly differentiated adenocarcinoma with Gleason score of 5 + 4 = 9 with DCa (Fig. 1). Magnetic resonance imaging showed local invasion of the left lobe of the prostate and bone metastasis of the left trochanteric section of the femur (Fig. 2). Although he was treated with androgen deprivation therapy (ADT), 10 months later, the disease became castration-resistant prostate cancer. At this point, the PSA level was 3.66 ng/mL and serum NSE was 9.6 ng/mL. Then, we administered three courses of docetaxel (75 mg/m^2^) as the second-line therapy. Since the serum PSA level was decreased to 0.54 ng/mL, the patient was treated with a combination of 160 mg of enzalutamide and intensity-modulated radiation therapy (IMRT) consisting of 78 Gy administered in 39 fractions for the prostate and 40 Gy administered in 14 fractions for the left femur as the third-line therapy, which decreased the PSA level to be nadir of 0.03 ng/mL. After the combination therapy, enzalutamide was continued for 13 months, and when the serum PSA increased to 0.35 ng/mL, four courses of cabazitaxel (25 mg/m^2^) were administered as the fourth-line therapy. Despite multimodal therapy, positron emission tomography/computed tomography (PET/CT) showed viability of the left trochanteric section of the femur. Thus, we performed left femoral head replacement, and we confirmed the absence of viable cancer cells pathologically. After 1 year, he was treated with local radiation of 60 Gy administered in 25 fractions because the PSA level increased to 0.76 ng/mL and pelvic lymph node metastasis was noted. At this point, we performed targeted genome sequencing of the tumor specimen from the transurethral resection and re-evaluated the immunochemistry findings. We identified *PTEN* p. R233fs*10, *RB1* loss exons 18–27, and *TP53* p.R249G mutations by genome sequencing (Table 1), and the patient was found to be positive for synaptophysin by immunohistochemistry (Fig. 1F). Currently, he is undergoing chemotherapy with cabazitaxel, and his PSA/NSE level was around 0.20/9.0 ng/mL.

**Table 1:** Results of FoundationOne® companion diagnostic (F1CDx) assay

Biomarker findings		Genomic findings	
MSI	Cannot be determined	PTEN	R233fs*10, splice site 480_492 + 2delCAGAGACAAAAAGGT
TMB	3Muts/Mb	RB1	Loss
		TP53	R249G

MSI, microsatellite instability; TMB, tumor mutational burden

## Discussion and conclusions

Patients with metastatic ACa are currently best treated with palliative drug treatments, such as ADT plus androgen receptor-targeted agents or chemotherapy [7]. Recent studies have demonstrated that early detection and aggressive treatment of metastatic lesions with surgery or radiation therapy appears to be a feasible strategy in patients with newly diagnosed oligometastatic ACa [8–10]. On the other hand, DCa has no established treatment strategy, and palliative ADT or chemotherapy alone cannot be expected to have a long-term prognosis even though a significantly greater frequency of men with DCa had local or distant metastasis at diagnosis. In our case, the patient was aggressively treated with ADT and chemotherapy with IMRT and local radiation therapy, while the initial diagnosis was a DCa with a Gleason score of 5 + 4 = 9 of ACa and bone metastasis. When PET/CT revealed revitalization of the left trochanteric section of the femur, we decided to perform the operation and confirmed the absence of viable cancer cells. Based on these aggressive multidisciplinary treatments made for the long-term survival.

Currently, in Japan, we can perform genome sequencing of tumor specimens in order to find the next treatment target drug in rare cancer patients in which the typical treatment effect becomes less remarkable. In this case, we identified *PTEN* and *RB1* co-loss and *TP53* mutations by next-generation sequencing. *PTEN* loss, *RB1* loss, and *TP53* mutations are common genomic alterations in many cancers. Vinceneux et al. reported that a complete loss of *PTEN* expression was more frequent in DCa (34%) than in ACa (11%) by immunohistochemistry [11]. Some reports have also indicated that *PTEN* loss occurring in DCa is associated with multiple markers of poor prognosis and has recently been associated with cribriform morphology, similar to that of ductal histology [12–14]. In fact, there were some scattered areas of complete loss of *PTEN* expression (Fig. 1C). Similarly, loss of function of the tumor suppressive function of *TP53* and *RB1* are the common mutant genes in ACa and are associated with aggressive tumor progression or metastatic ACa [15–17], while there was no confident evidence about the correlation between *RB1* or *TP53* mutation and DCa. Tarjan et al. reported that the combination of *TP53* and chromogranin A expression in cancer cells is associated with ductal differentiation of ACa [18]. On the other hand, we performed immunohistochemistry for chromogranin A and synaptophysin staining, and the patient was found to be positive for synaptophysin (Fig. 1F) because these three genomic mutations are often found in patients with NEPC [19]. These results indicated that we could have the treatment option to perform the administration of a platinum agent for the future.

In conclusion, to the best of our knowledge, there are few case reports of genomic analysis for DCa, which is the first Japanese report of DCa with *PTEN* and *RB1* co-loss and *TP53* mutation. Genetic analysis may help not only guide the therapeutic strategies, but also sometimes with the diagnosis.

## Data Availability

The datasets used and analyzed in this study are available from the corresponding author on reasonable request.

## Abbreviations

DCa: Ductal adenocarcinoma
ACa: Acinar adenocarcinoma
NEPC: Neuroendocrine prostate cancer
PSA: Prostate-specific antigen
ADT: Androgen deprivation therapy
IMRT: Intensity modulated radiation therapy
PET/CT: Positron emission tomography/ computed tomography
